# Supplementary material for: Lithium crystallization at solid interfaces
Source: Nat Commun. 2023 May 24;14:2986. doi: 10.1038/s41467-023-38757-2 (PMC10209073; doi:10.1038/s41467-023-38757-2)
Supplement: Supplementary file 1 — Supplementary Information File [file 41467_2023_38757_MOESM1_ESM.pdf]

Supporting Information

Lithium Crystallization at Solid Interfaces

Menghao Yang[1], Yunsheng Liu[1], Yifei Mo[1, 2 *]

1. Department of Materials Science and Engineering, University of Maryland, College Park, MD, USA.
2. Maryland Energy Innovation Institute, University of Maryland, College Park, MD, USA.

[*] Email: yfmo@umd.edu.

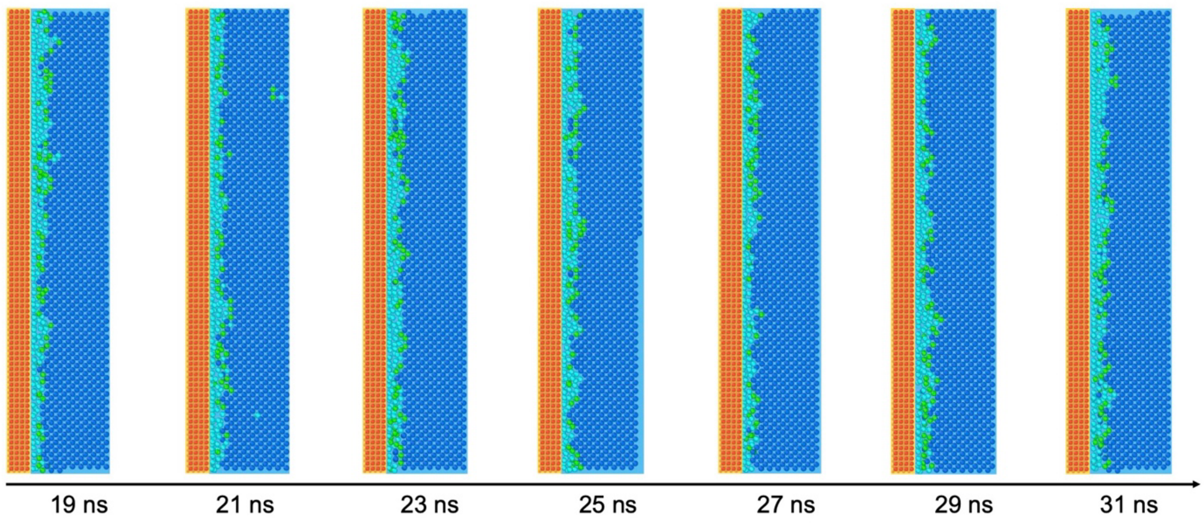

Supplementary Figure 1. The atomistic structures of the Li–SE interface over time during Li insertion. Side view of the Li metal slab with a thickness of 0.5 nm. Li atoms in Li metal are classified as BCC- (Blue), FCC- (Green), and disordered- (Cyan) Li atoms.

Supplementary Note 1. The Li insertion potential.

Since the energy $E(t)$ of the Li metal slab was dependent on the number of Li atoms $N(t)$ at time t, we plotted (Fig. 1, Fig. 4 and Supplementary Fig. 2a) the energy of Li metal referenced to crystalline bulk Li E_{bulk} per interface area A defined as follows:

$$\Delta E(t) = \frac{E(t) - E_{bulk} \times N(t)}{A}, \quad \text{(S1)}$$

where E_{bulk} was the average per-atom energy of perfect bulk crystalline Li metal obtained from MD simulations at 300 K. The energy showed a period of 8 ns corresponding to the insertion and transformation of an atomistic layer of Li metal (Supplementary Fig. 2a, and Fig. 1).

The potential to insert Li was evaluated as follows. A total number of $N(t)$ Li atoms was inserted at time t. To insert $N(t+\Delta t) - N(t)$ Li atoms during a short time interval Δt, the potential (referenced to bulk Li metal) was evaluated as:

$$\phi_{\Delta t}(t) = \frac{E(t+\Delta t) - E(t)}{N(t+\Delta t) - N(t)} - E_{bulk} \quad \text{(S2)}$$

For a short time interval Δt, this potential $\phi_{\Delta t}(t)$ can be understood as instantaneous potential to insert Li at time t, as shown in Supplementary Fig. 2b and Supplementary Fig. 16 for $\Delta t = 0.5$ ns. During Li insertion, for each period, the instantaneous potential $\phi_{\Delta t}$ was positive during the early time of energy period due to the increased energy, with a peak value in the range of 22 to 38 meV (Supplementary Fig. 2b).

The average potential of Li insertion from initial time t_0 to time t can be evaluated as

$$\phi_{t_0}(t) = \frac{E(t) - E(t_0)}{N(t) - N(t_0)} - E_{bulk} \quad \text{(S3)}$$

This average potential $\phi_{t_0}(t)$ was equivalent to the average value of instantaneous potential $\phi_{\Delta t}$ over the time period from time t_0 to time t. In Supplementary Fig. 2c and Supplementary Fig. 17, the averaged potential $\phi_{t_0}(t)$ were plotted with t_0 set to the bottom of each energy period from Fig.

1. During Li insertion, the average potential ϕ_{t_0} had the maximum value in the range of 18 to 35 meV for the first four periods (Supplementary Fig. 2c). Since the equilibrium potential is Li bulk, these potentials were equivalent to the overpotential for Li crystallization during Li deposition.

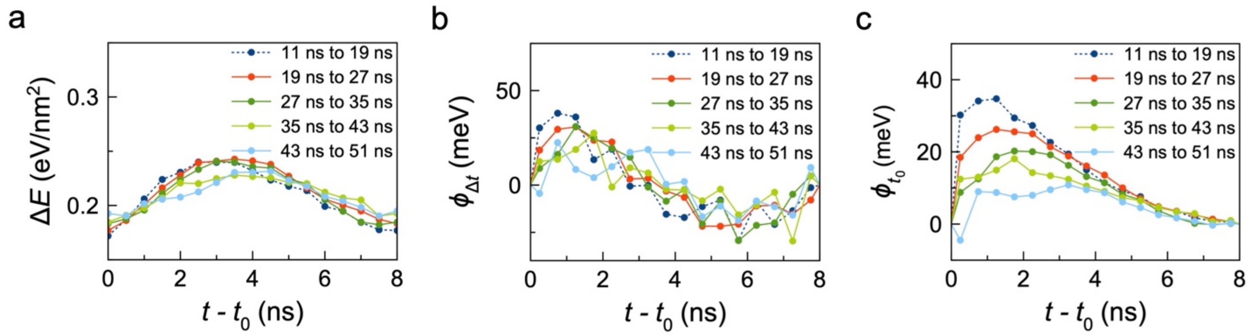

Supplementary Figure 2. a) The energy of Li metal referenced to crystalline bulk Li E_{bulk} per interface area A, **b)** instantaneous potential $\phi_{\Delta t}$, and **c)** average potential ϕ_{t_0} for each period starting at the period-bottom time t_0.

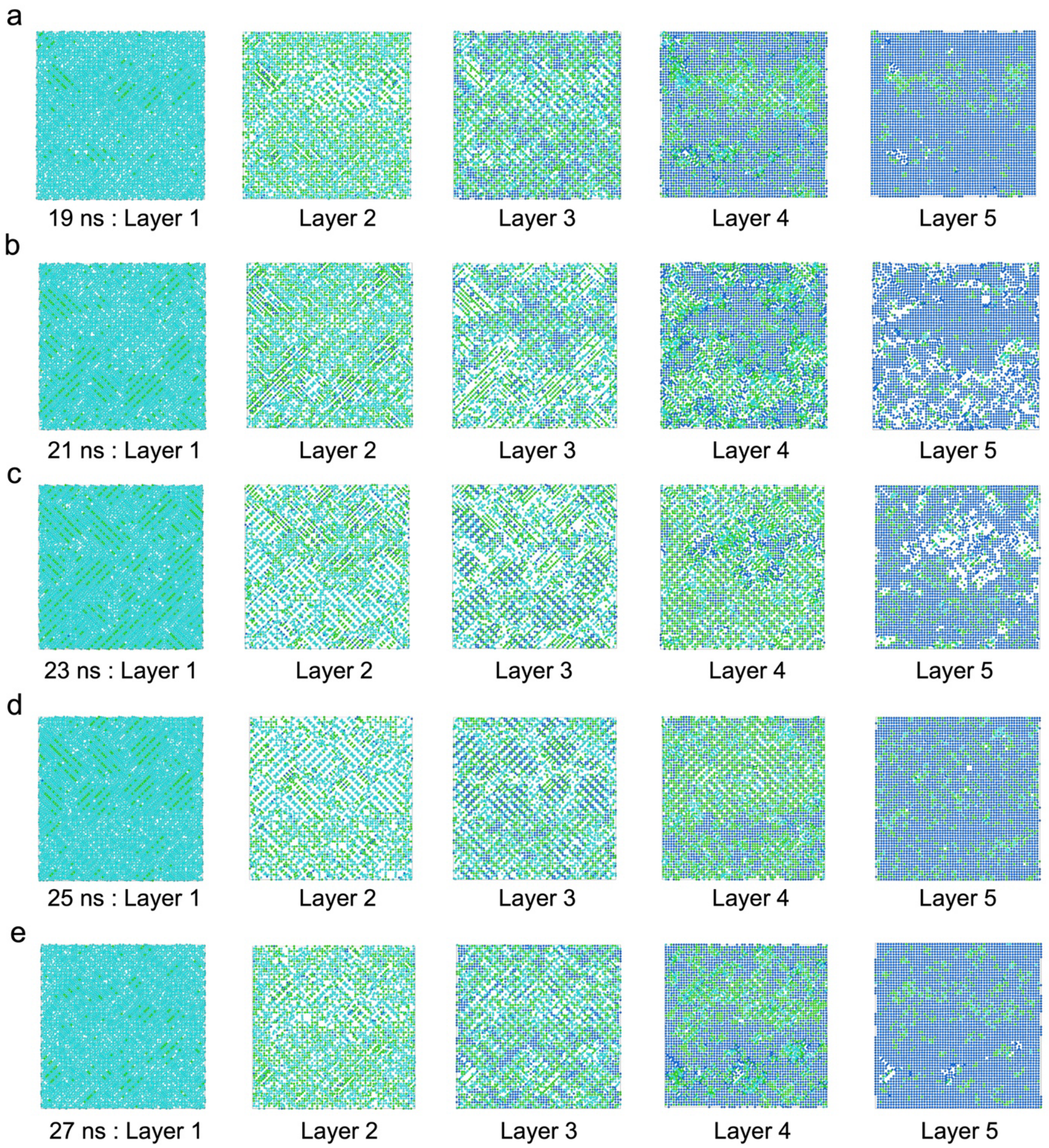

Supplementary Figure 3. Interfacial atomistic structures at the Li–SE interfaces with layer-by-layer bottom view at **a)** 19 ns, **b)** 21 ns, **c)** 23 ns, **d)** 25 ns, **e)** 27 ns. The 1st layer is 2.2 Å to the SE, and each layer beyond is 1.75 Å in thickness.

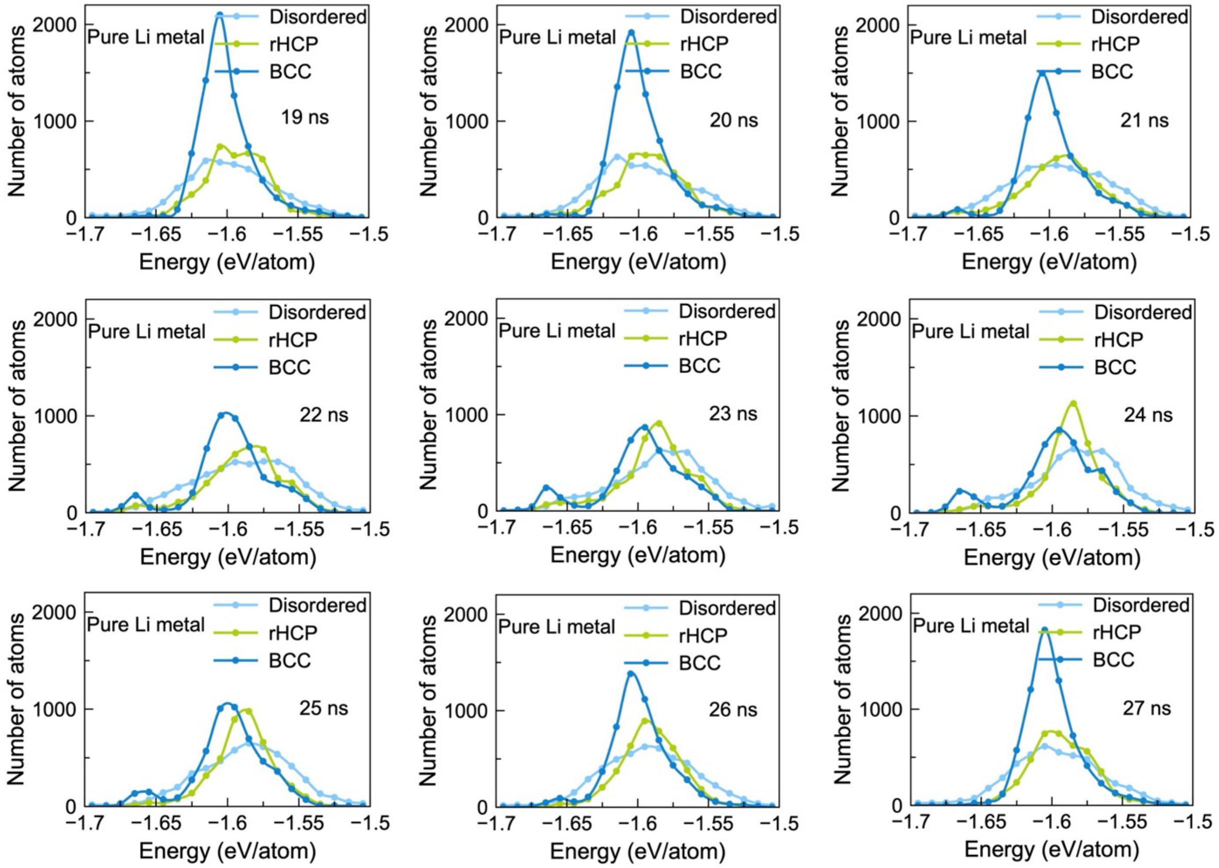

Supplementary Figure 4. The Li density of atomistic states (DOAS) showing the statistics of the atomistic energies of different Li types (disordered, rHCP and BCC) in the 7.0 Å-thick layer (2nd to 5th layers) for the pristine Li–SE interface at different deposition times.

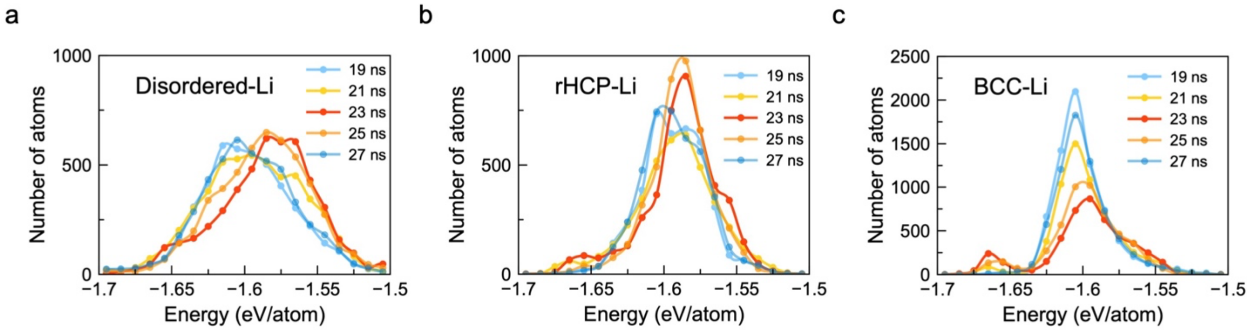

Supplementary Figure 5. The Li density of atomistic states (DOAS) showing the statistics of the atomistic energies of different Li types, **a)** disordered, **b)** rHCP, and **c)** BCC, in the 7.0 Å-thick layer (2nd to 5th layers) for the pristine Li–SE interface at different deposition times.

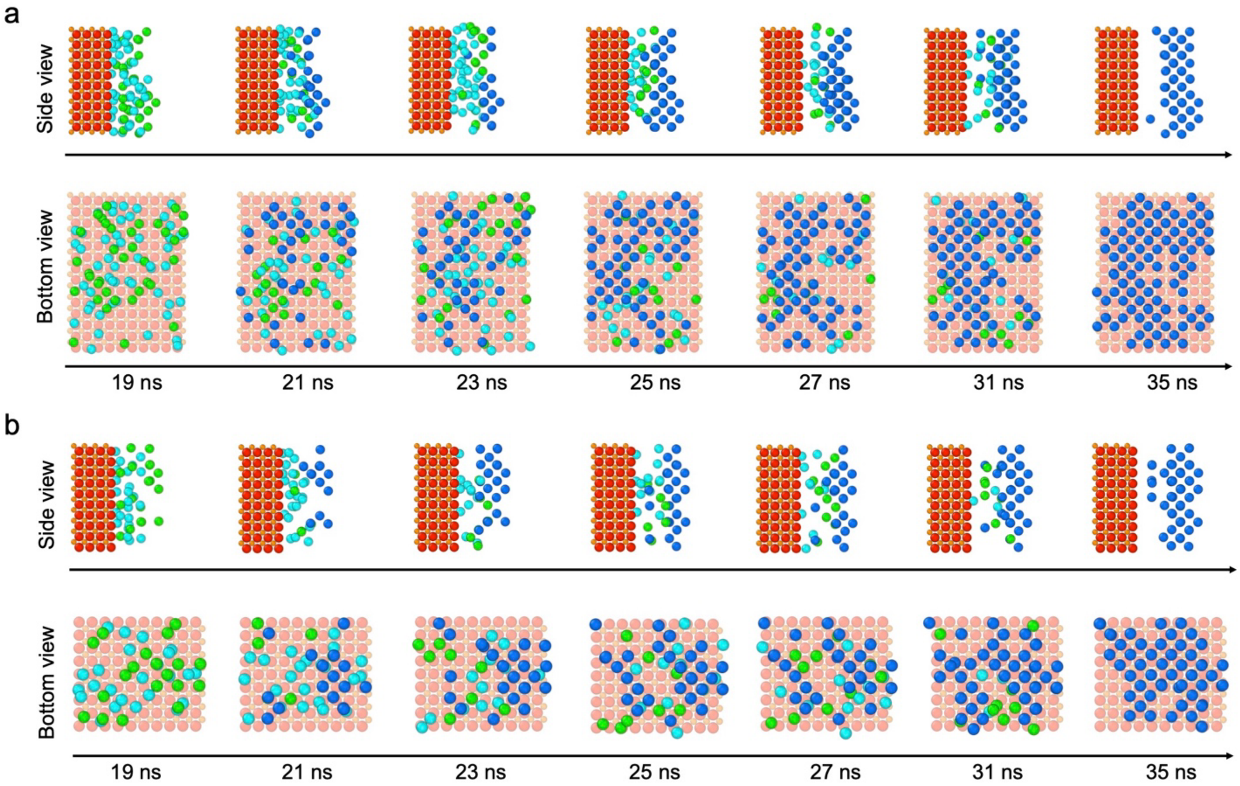

Supplementary Figure 6. Crystallization process of a group of Li atoms for **a)**–**b)** two different atomistic clusters from non-BCC-Li clusters.

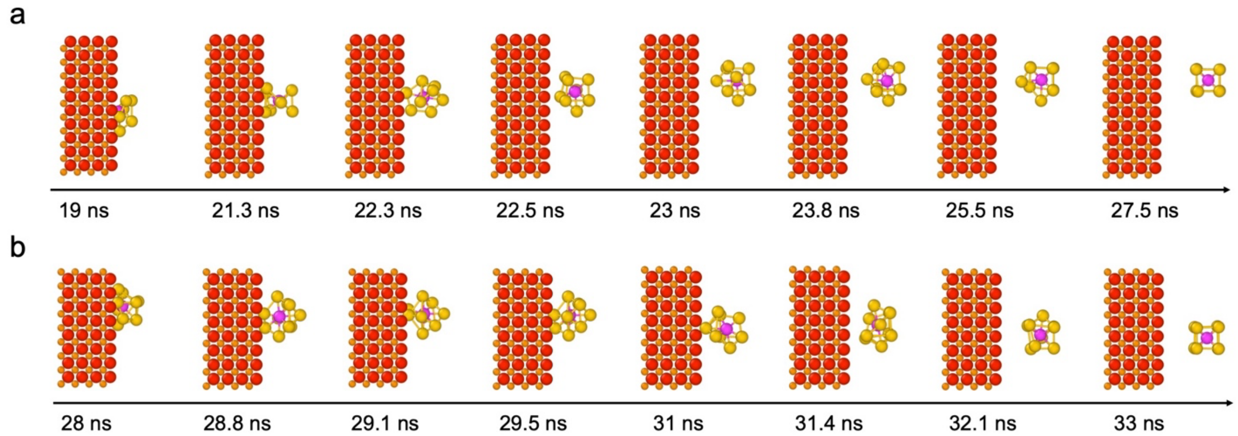

Supplementary Figure 7. Crystallization process of **a)**–**b)** two different inserted Li atom (purple) and its neighboring Li (orange).

Supplementary Note 2. Li metal interfaces with other materials.

In addition to the Li_2O interface in the main text, we constructed atomistic models of Li–SE interfaces by using Li metal slab with (100) surface in contact with (100) surface of $Li_7La_3Zr_2O_{12}$ (LLZO) SE and LiF SE. The same scheme of the interatomic potential was used for LiF and LLZO as for Li_2O (Methods). The parameters were listed in Supplementary Table 2 and were adopted from the recent study (*1*). The repulsion parameters for F^-–Li were adopted from Ref. (*2*). The values of A_{ij} for La^{3+}–Li and Zr^{4+}–Li repulsions are set to three and four times that of Li^+–Li repulsion interactions. For the Li(100)–LiF(100) interface, the interfacial adhesion was calculated to be 0.23 J/m^2, in comparison to 0.1 J/m^2 from DFT calculations (*3*). For the Li(100)–LLZO(100) interface, the interfacial adhesion was calculated to be 0.77 J/m^2, which agreed well with 0.67–0.98 J/m^2 of Li-LLZO interfaces from DFT calculations (*4, 5*).

Large-scale MD simulations were performed to reveal the interface structures and Li crystallization processes at the Li(100)–LLZO(100) and Li(100)–LiF(100) interfaces, respectively. Due to the large lattice mismatch between Li metal and LLZO SE, an interfacial amorphous lithium layer is formed at Li(100)–LLZO(100) interfaces. The interfacial amorphous layer exhibits local configurations of rHCP- or disordered-Li atoms. As shown in Supplementary Fig. 9, most of Li atoms of the first Li layer in contact with LLZO SE (2.20 Å from the LLZO SE interface) are identified as disordered-Li atoms. Further from Li(100)–LLZO(100) interfaces, the second to the fifth Li layers (2.20 Å – 9.20 Å from the LLZO SE interface) contain more rHCP-Li, especially in the third (3.95 Å – 5.70 Å from the LLZO SE interface) and fourth Li layer (5.70 Å – 7.45 Å from the LLZO SE interface). The energy barrier of Li crystallization at Li(100)–LLZO(100) also shows a periodicity of 8 ns with peak energies of 0.83 – 0.87 eV/nm^2 and energy minima of 0.67 eV/nm^2, indicating a total barrier of 0.16 – 0.20 eV/nm^2. This periodic energy profile of Li deposition is

related to the energy barrier of Li metal crystallization process for the Li layer in contact with LLZO SE, similar to that with Li_2O (Fig. 1-3). In addition, the trends of disordered-Li (Supplementary Fig. 10b) and rHCP-Li (Supplementary Fig. 10c) are also closely correlated with the energy of the Li metal-LLZO SE interface (Supplementary Fig. 10a), similar to Li_2O interfaces, confirming the critical role of disordered-Li and rHCP-Li in the energy barrier of Li crystallization.

The interfacial atomistic structures near Li(100)–LiF(100) interfaces also exhibit local configurations of rHCP-Li or disordered-Li atoms. The first two Li layers in contact with LiF SE (4.15 Å from the LiF SE interface in Supplementary Fig. 11) are consisting of most disordered-Li atoms. By calculating the energy barrier of Li crystallization at Li(100)–LiF(100) interface, the energy excess to the equilibrium bulk Li per interfacial area fluctuates with a periodicity of 8 ns with peak energies of 2.31 – 2.35 eV/nm^2 and energy of 2.06 – 2.17 eV/nm^2, suggesting the energy barrier of 0.17 – 0.29 eV/nm^2. The periodic energy of Li metal–LiF SE (Supplementary Fig. 12a) is related to the trends of disordered-Li (Supplementary Fig. 12b) or rHCP-Li atoms (Supplementary Fig. 12c), indicating the crystallization and growth of a full atomistic layer at the interface of the Li metal slab in contact with LiF SE. Therefore, the Li crystallization process and related energy barrier is general for a range of SE interfaces with different SE materials.

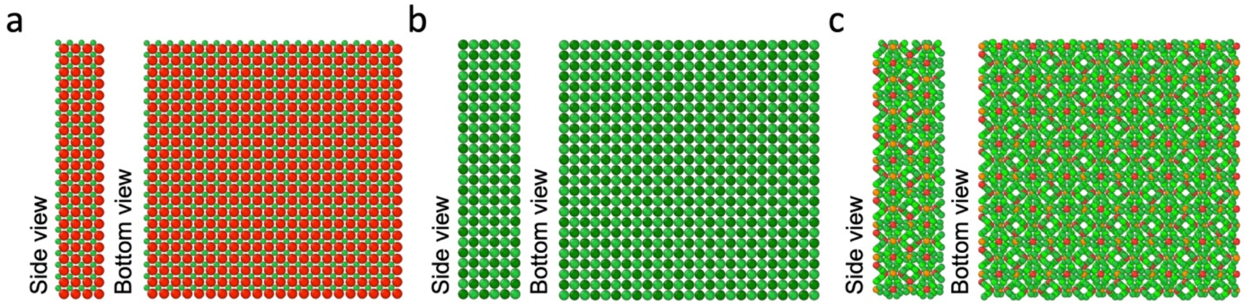

Supplementary Figure 8. Atomistic structures of the (100) surface termination for **a)** Li_2O (Li^+: green, O^{2-}: red), **b)** LiF (F^-: dark green), **c)** LLZO (La^{3+}: red, Zr^{4+}: orange, O^{2-}: light green). All surface terminations are stoichiometric and are constructed by cutting the face of the unit cell.

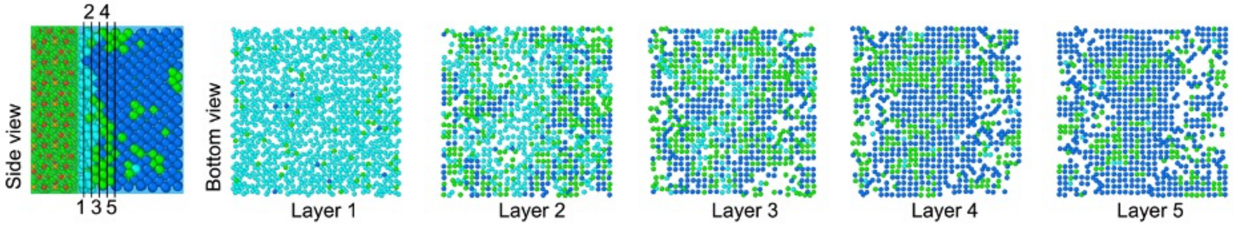

Supplementary Figure 9. Interfacial atomistic structures at Li(100)–LLZO(100) interfaces at 5 ns with layer-by-layer bottom view. The 1st layer is within 2.2 Å from LLZO SE, and each layer beyond is 1.75 Å in thickness. Disordered-, rHCP-, and BCC-Li are shown in cyan, green, and blue, respectively.

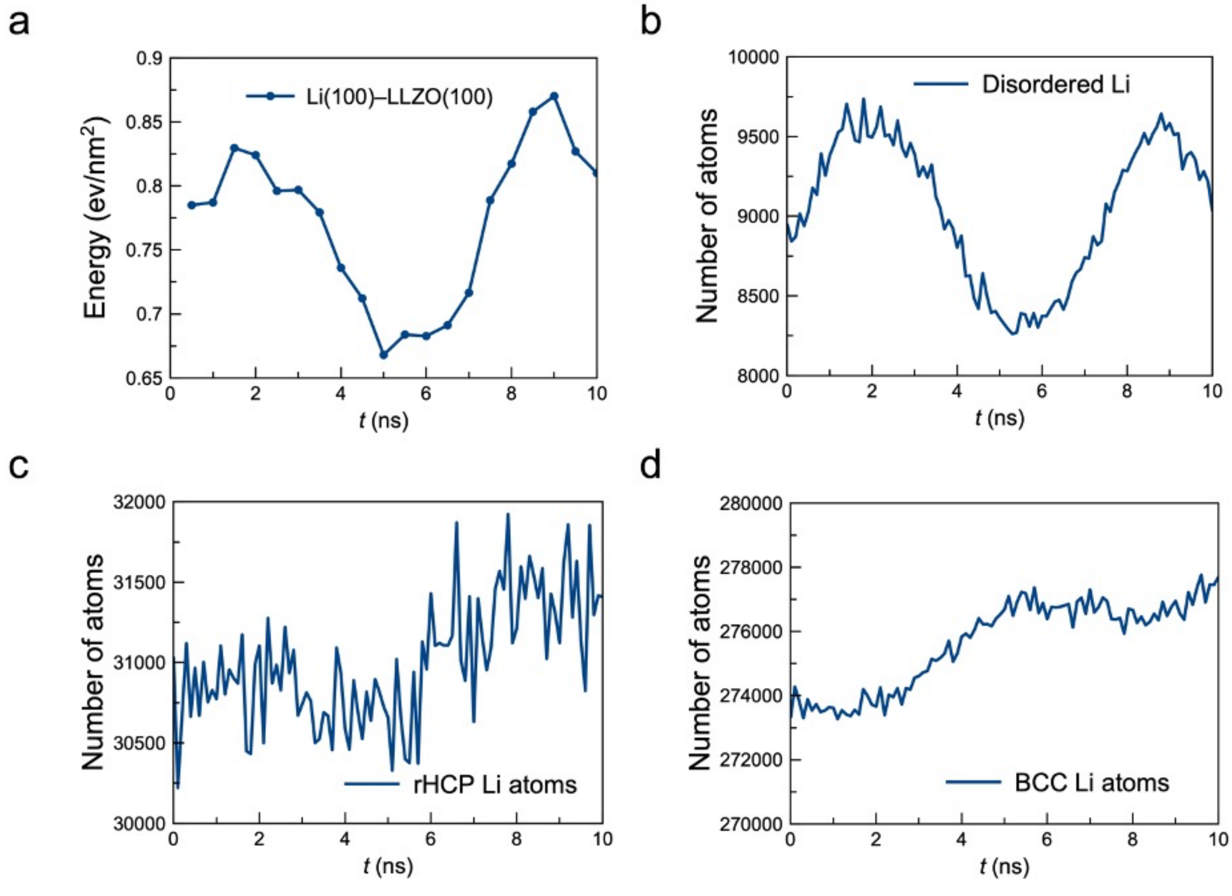

Supplementary Figure 10. Li crystallization at engineered Li(100)–LLZO(100) interfaces. a) The energy of Li metal referenced to crystalline bulk Li per area and the number of Li atoms with different local configurations of **b)** Disordered, **c)** rHCP, **d)** BCC, during the Li insertion. The solid markers in **a)** are 500 ps-averaged energies.

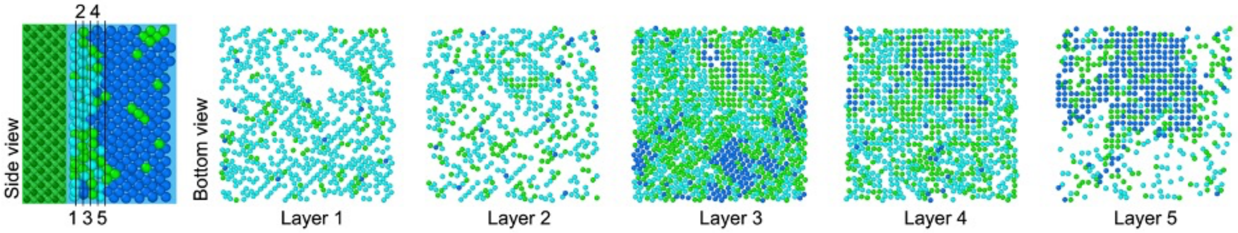

Supplementary Figure 11. Interfacial atomistic structures at Li(100)–LiF(100) interfaces at 5 ns with layer-by-layer bottom view. The 1st layer is within 2.4 Å from LiF SE, and each layer beyond is 1.75 Å in thickness.

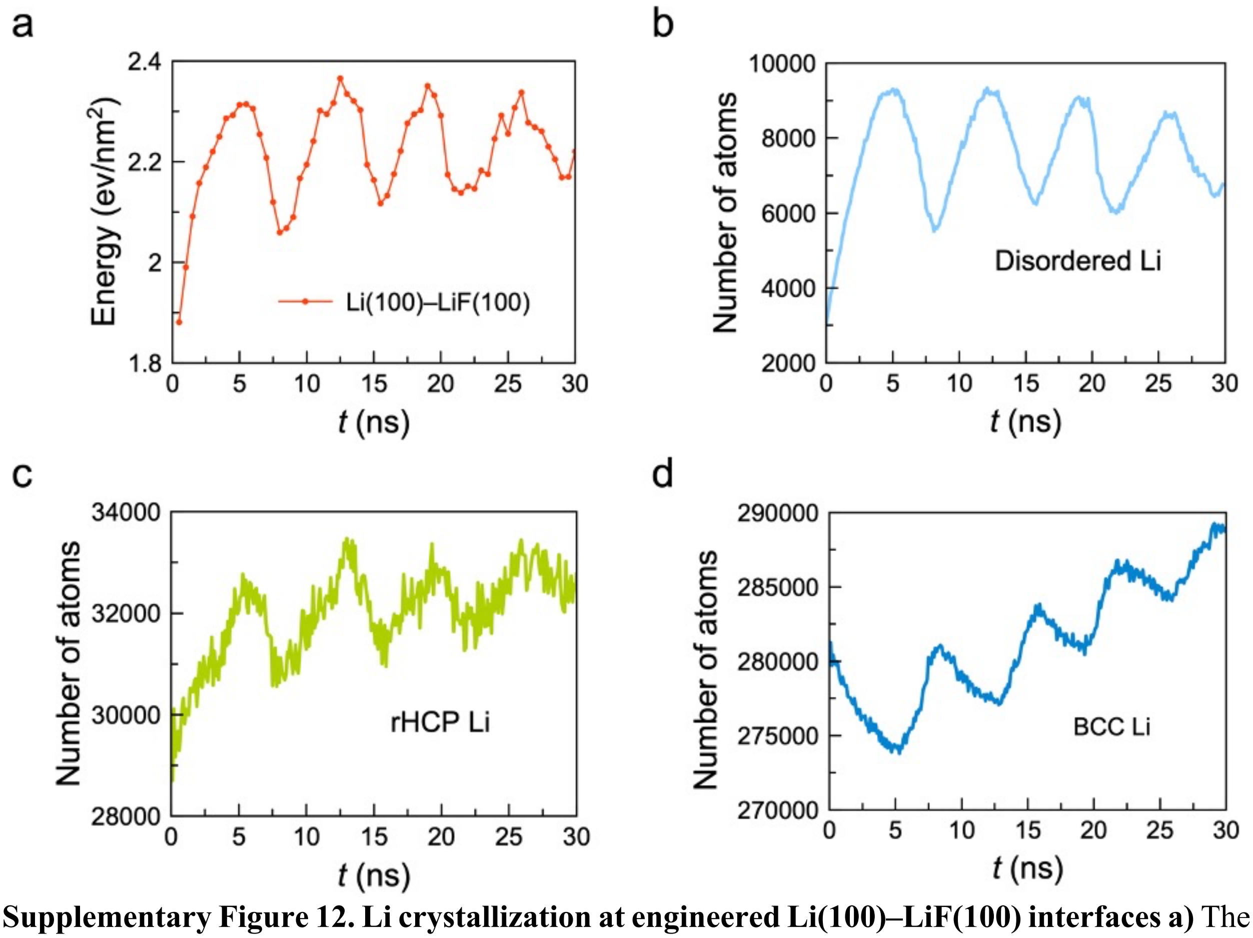

Supplementary Figure 12. Li crystallization at engineered Li(100)–LiF(100) interfaces a) The energy of Li metal referenced to crystalline bulk Li per area and the number of Li atoms with different local configurations of **b)** Disordered, **c)** rHCP, **d)** BCC, during the Li insertion. The solid markers in **a)** are 500 ps-averaged energies.

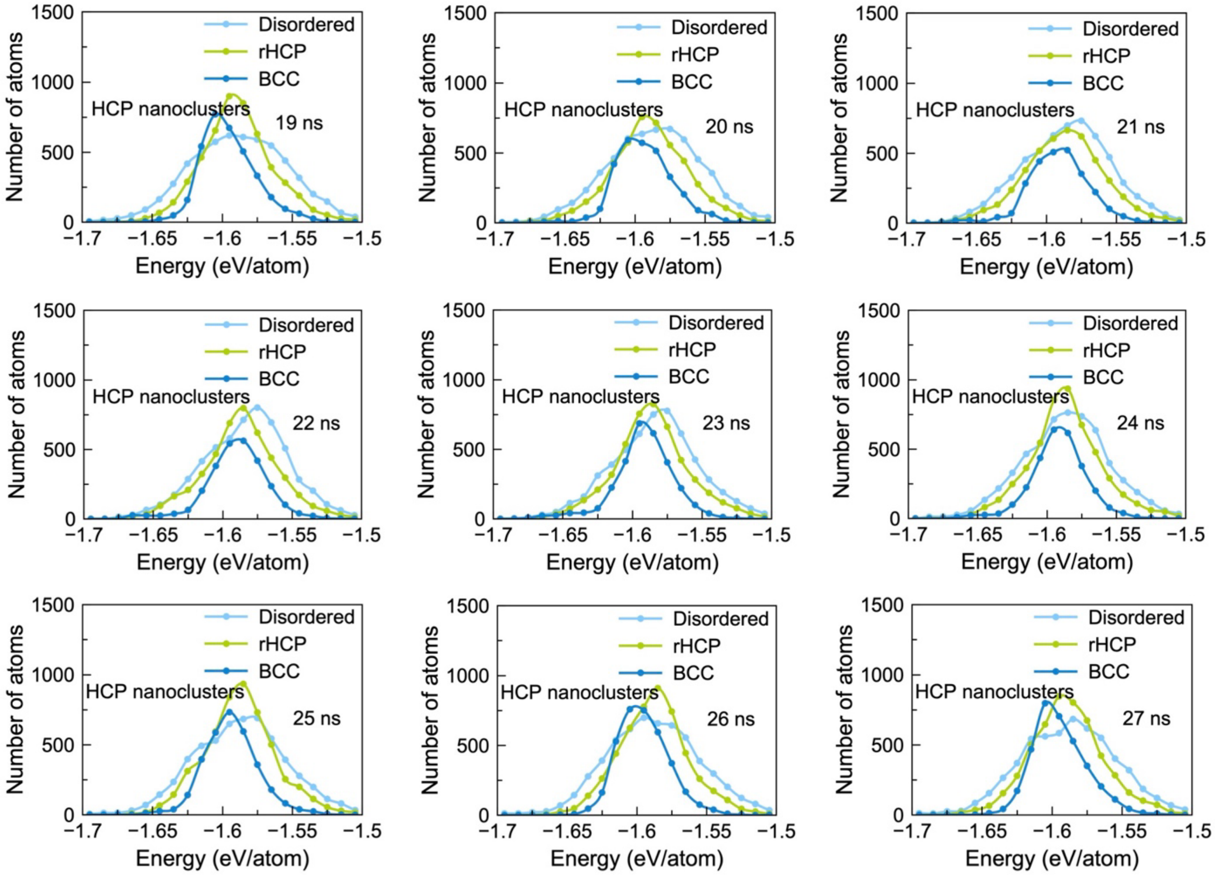

Supplementary Figure 13. The Li density of atomistic states (DOAS) showing the statistics of the atomistic energies of different Li types (disordered, rHCP and BCC) in the 7.0 Å-thick layer (2nd to 5th layers) for the Li–SE interface with HCP-Li nanoclusters at different deposition times. For this Li–SE interface with HCP nanoclusters, the atomistic energies of rHCP-Li are generally lower than those of disordered-Li at all deposition times, in comparison to the pristine Li–SE interface.

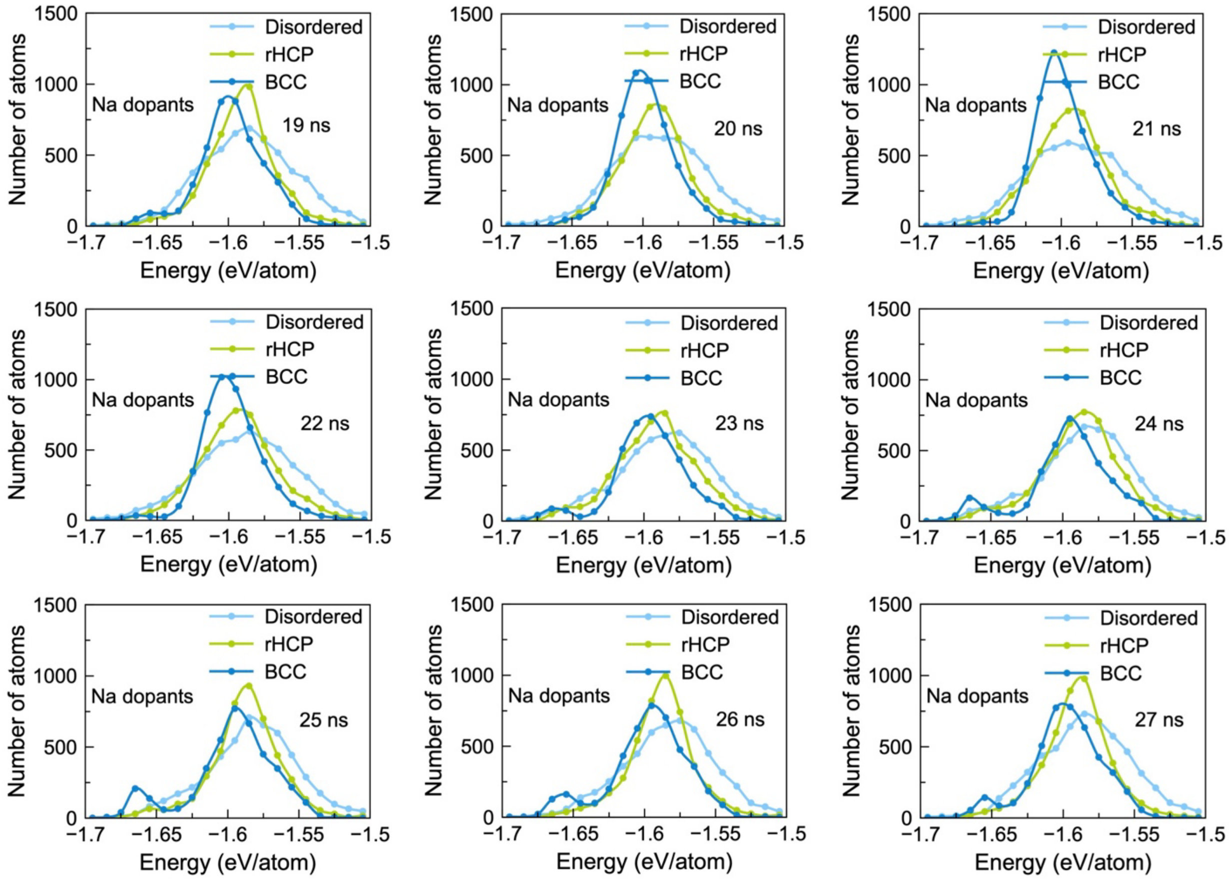

Supplementary Figure 14. The Li density of atomistic states (DOAS) showing the statistics of the atomistic energies of different Li types (disordered, rHCP and BCC) in the 7.0 Å-thick layer (2nd to 5th layers) for the Li–SE interface with Na dopants at different deposition times. For this Li–SE interface with Na dopants, the atomistic energies of rHCP-Li are generally lower than those of disordered-Li at all deposition times, in comparison to the pristine Li–SE interface.

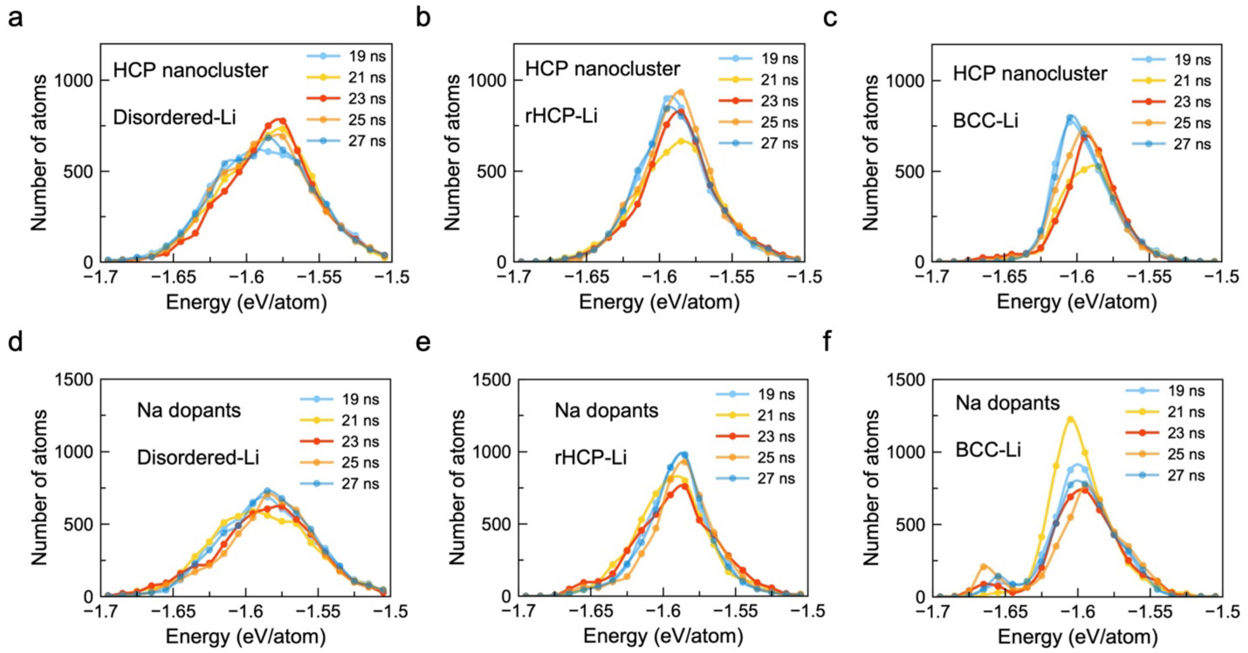

Supplementary Figure 15. The Li density of atomistic states (DOAS) showing the statistics of the atomistic energies of different Li types, disordered, rHCP, and BCC (from left to right) in the 7.0 Å-thick layer (2nd to 5th layers) for Li–SE interfaces with **a)**–**c)** HCP-Li nanoclusters and **d)**–**f)** Na dopants.

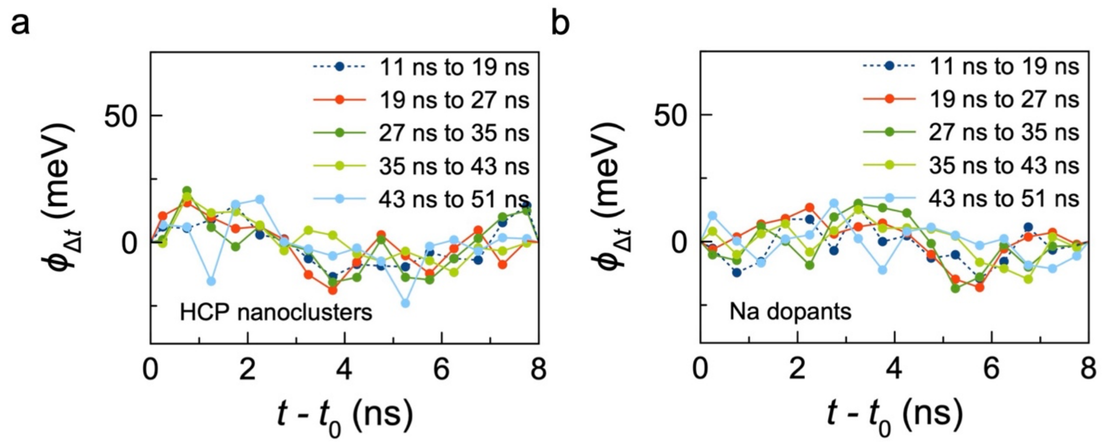

Supplementary Figure 16. Instantaneous potential $\phi_{\Delta t}$ of Li insertion for the Li–SE interface with **a)** HCP nanoclusters and **b)** Na dopants for each energy period.

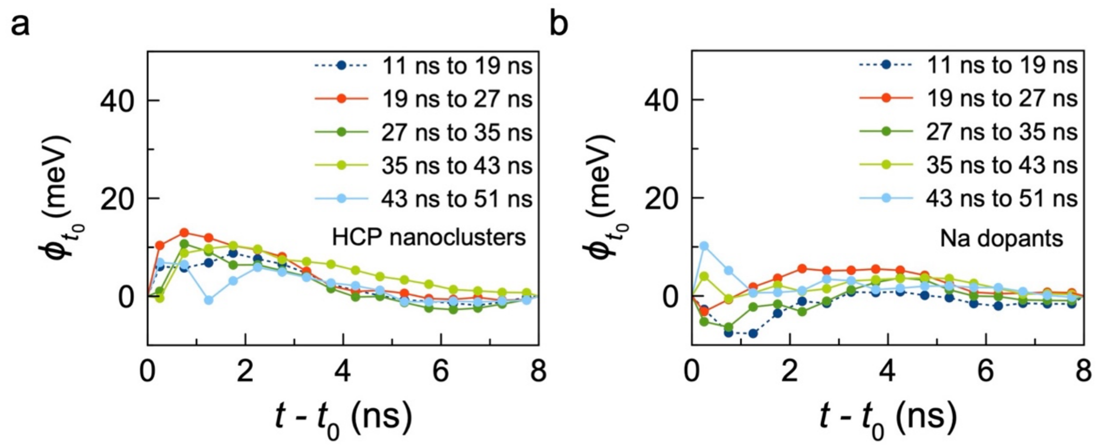

Supplementary Figure 17. Average potential ϕ_{t_0} of Li insertion for the Li–SE interface with **a)** HCP nanoclusters and **b)** Na dopants at each period with t_0 set to the bottom of each energy period.

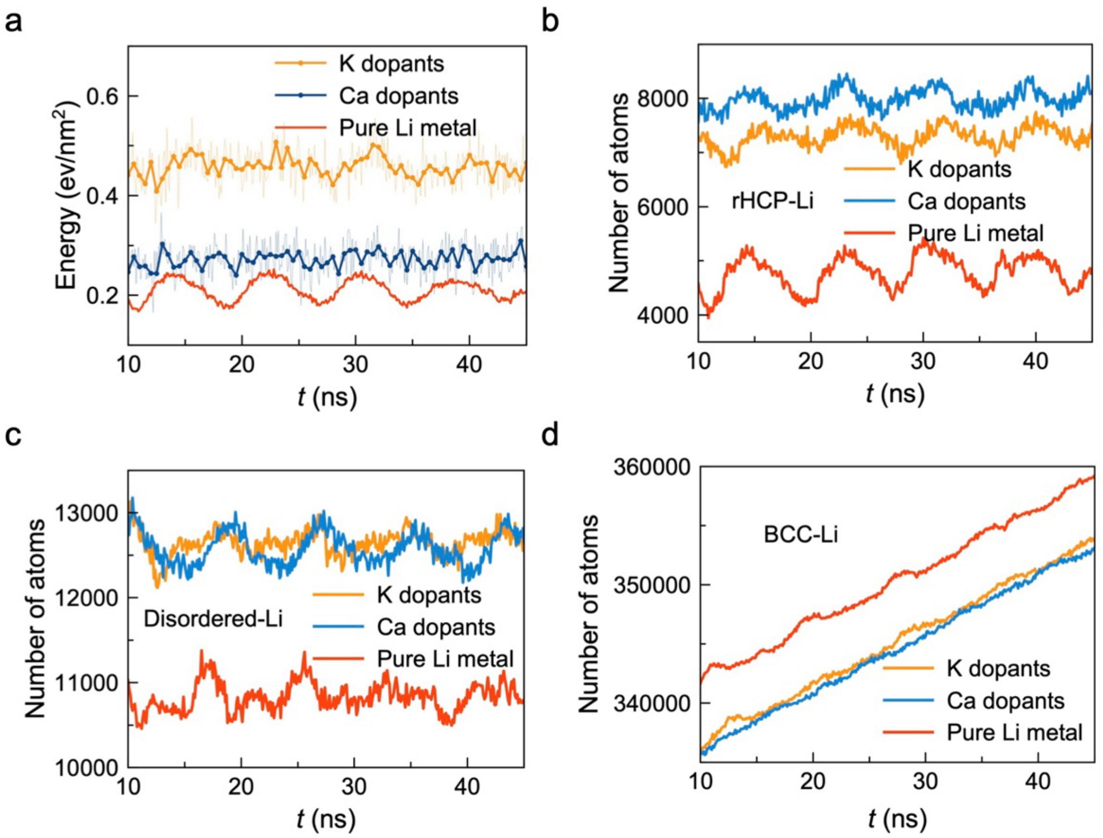

Supplementary Figure 18. a) The energy of Li metal referenced to crystalline bulk Li per area and the number of Li atoms with different local configurations **b)** rHCP, **c)** Disordered, **d)** BCC, during the Li insertion with pristine Li–SE interface (red), interface with K dopant (orange), and interface with Ca dopant (blue). The placement and distribution of K and Ca dopants in these interfaces are identical to those of the Na dopants described in the Methods. The light and solid markers in **a)** are instantaneous and 500ps-averaged energies, respectively.

Supplementary Table 1. Calculated physical properties for bulk lithium phrases. Lattice constants a_0 and c_0 in Å, elastic constants (C_{11}, C_{12}, and C_{44}) and bulk modulus B_0 in GPa, surface energies γ in J/m^2, cohesive energies E_{coh} and vacancy formation energies E_{vac} in eV/atom, diffusion coefficients D_{Li} in 10^{-11}cm^2/s, the energy of FCC/HCP phase (E_{FCC}/E_{HCP}) above the BCC phase in eV/atom.

		Classical potential (*6*)	DFT (*7*)	Experimental values
BCC	a_0	3.51	3.43	3.49 (*8*)
	C_{11}	20	15	13.42 (*9*)
	C_{12}	12	13	11.30 (*9*)
	C_{44}	10	11	8.89 (*9*)
	B_0	14.5	14	13.3 (*10*), 12.0 (*9*)
	γ_{100}	0.29	0.46	
	γ_{110}	0.33	0.50	0.522 (*11*), 0.525 (*12*)
	γ_{111}	0.41	0.54	
	E_{coh}	1.65	1.61 (*13*), 1.68 (*13*)	1.69 (*14*)
	E_{vac}	0.54	0.53 (*15*)	
	D_{Li}	3.05	1 – 10 (*16*)	5 – 9 (*17*)
FCC	a_0	4.55	4.32	
	E_{FCC}	0.023	-0.002	
	C_{11}	22	-5	
	C_{12}	11	23	
	C_{44}	16	10	
	B_0	18	14	
	γ_{100}	0.38	0.47	
	γ_{110}	0.39	0.54	
	γ_{111}	0.60	0.50	
HCP	a_0	3.22	3.08	3.11 (*8*)
	c_0	5.26	4.92	5.09 (*8*)
	E_{HCP}	0.023	-0.002	
	C_{11}	29	22	
	C_{12}	7	11	
	C_{44}	19	6	
	B_0	24	14	
	γ_{0001}	0.70	0.53	
	$\gamma_{10\text{-}10}$	0.45	0.53	
	$\gamma_{11\text{-}20}$	0.32	0.51	

Supplementary Table 2. Short-range potential parameters for Li_2O, LiF and LLZO(100) with Li metal.

Pairs	A_{ij} (eV)	ρ (Å)
Li_2O: Li^+–Li	465.54	0.2939
LiF: Li^+–Li	2575.00	0.26
LLZO: La^{3+}–Li	1396.62	0.2939
LLZO: Zr^{4+}–Li	1862.16	0.2939
	ϵ (eV)	r_m (Å)
Li_2O: O^{2-}–Li	0.273	2.00
LiF: F^-–Li	0.198	2.04
LLZO: O^{2-}–Li	0.287	1.90

Supplementary Table 3. The Lennard-Jones potential parameters for dopants (see Methods).

ε (eV)		σ (Å)	
$\varepsilon_{Na\text{-}Li}$	0.168	$\sigma_{Na\text{-}Li}$	3.157
$\varepsilon_{Ca\text{-}Li}$	0.210	$\sigma_{Ca\text{-}Li}$	3.220
$\varepsilon_{K\text{-}Li}$	0.153	$\sigma_{K\text{-}Li}$	3.562

Supplementary Note 3. Dopant effect on energies.

We here test the effect of Na, Ca, and K dopants on the energies and configurations of Li atoms. As described in Methods, the potential parameters for Li-*M* (Na, Ca and K) interactions were listed in Supplementary Table 3. In the BCC Li metal with a single dopant *M*, we calculated the Li-*M* bond length and Li-Li bond length for those nearest-neighbor Li atoms of the dopant. The Li-M and nearby Li-Li bond length under the presence of M dopant show good agreement between DFT and the classical potentials (Supplementary Table 4).

We conducted the following test to verify the effect of dopant on the energies of Li atoms, which is relevant for the intermediate steps of the crystallization pathway. We constructed supercells of BCC and HCP Li metal with a total of 128 and 108 atoms, respectively. For each supercell, one Li atom was replaced by a Na, Ca and K dopant. To generate a range of atomic configurations mimicking those in the Li-SE interface, we performed MD simulations for 100 ps at 300 K in the NPT ensemble, and selected 20 configuration snapshots (1 ps each) from the last 20 ps for both Li phases. For all these snapshots of atomic configurations with a dopant in BCC and HCP Li, we evaluated and compared the energies predicted by the interatomic potentials and DFT calculations without static relaxations. The energies for every configuration are compared in Supplementary Fig. 20 and for the distribution in Supplementary Fig. 19. For most configurations of BCC and HCP Li under Na, Ca, and K dopant, there are strong correlations between the energies

of DFT and interatomic potentials (Supplementary Fig. 20). The distribution of all configurations also shows the effect of the dopant on the energies of BCC and HCP Li agree between interatomic potential and DFT.

Supplementary Table 4. Comparing Li-*M* dopant and Li-Li bond length for DFT and classical potential. The percentage number in parentheses shows the deviation from the original Li-Li bond in Li metal.

	**Li-*M* bond length (Å)**		**Li-Li bond length (Å) near *M* dopant**	
	DFT	potential	DFT	potential
Na	3.22 (8.3 %)	3.33 (9.7 %)	3.71 (8.3 %)	3.85 (9.7 %)
Ca	3.29 (10.6 %)	3.40 (11.7 %)	3.79 (10.6 %)	3.92 (11.7 %)
K	3.42 (15.2 %)	3.71 (22.0 %)	3.95 (15.2 %)	4.23 (20.4 %)

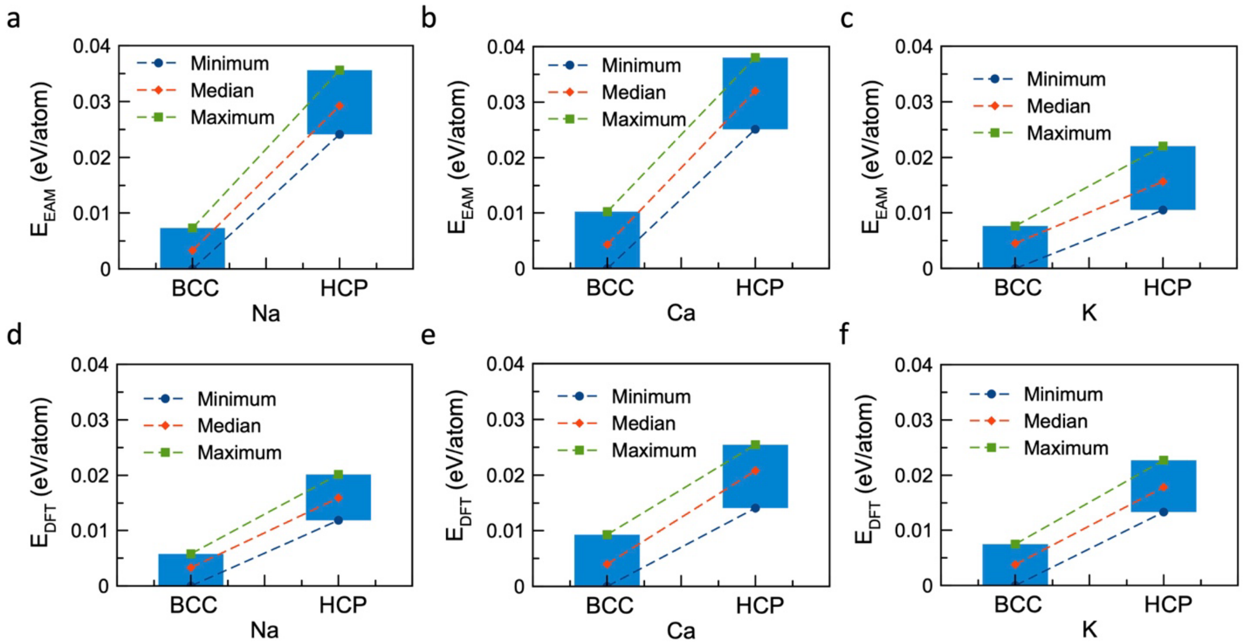

Supplementary Figure 19. The energy distributions of BCC and HCP Li supercells with a single **a)** Na, **b)** Ca, and **c)** K dopant atom by interatomic potentials, and BCC and HCP Li supercells with a single **d)** Na, **e)** Ca, and **f)** K dopant atom by DFT calculations. All energies are referenced to the lowest energy configuration of the BCC Li with a dopant.

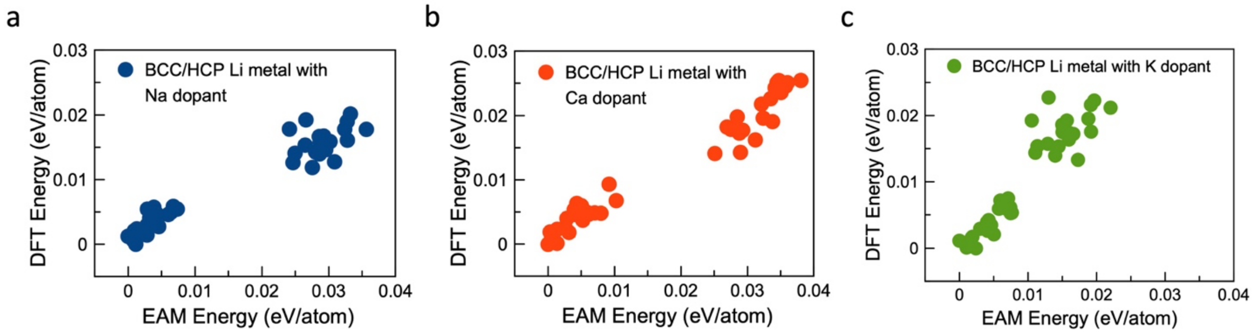

Supplementary Figure 20. Comparing the energy for every configuration of BCC and HCP Li with a single **a)** Na, **b)** Ca, and **c)** K dopant by interatomic potential and DFT. All energies are referenced to the lowest energy configuration of the BCC Li with a dopant.

Supplementary References

1. M. Yang, Y. Mo, Interfacial Defect of Lithium Metal in Solid-State Batteries. *Angew. Chem. Int. Ed.* **60**, 21494-21501 (2021).
2. T. R. Stechert, *Glasses for Energy Applications: Atomic Scale Network Structure and Properties*. (Imperical College London, 2013).
3. Z. Liu *et al.*, Interfacial Study on Solid Electrolyte Interphase at Li Metal Anode: Implication for Li Dendrite Growth. *J. Electrochem. Soc.* **163**, A592-A598 (2016).
4. B. Gao, R. Jalem, Y. Tateyama, Surface-Dependent Stability of the Interface between Garnet Li7La3Zr2O12 and the Li Metal in the All-Solid-State Battery from First-Principles Calculations. *ACS Appl. Mater. Interfaces* **12**, 16350-16358 (2020).
5. A. Sharafi *et al.*, Surface Chemistry Mechanism of Ultra-Low Interfacial Resistance in the Solid-State Electrolyte Li7La3Zr2O12. *Chem. Mater.* **29**, 7961-7968 (2017).
6. A. Nichol, G. J. Ackland, Property trends in simple metals: An empirical potential approach. *Phys. Rev. B* **93**, 184101 (2016).
7. A. Jain *et al.*, Commentary: The Materials Project: A materials genome approach to accelerating materials innovation. *APL Mater.* **1**, 011002 (2013).
8. C. S. Barrett, X-ray study of the alkali metals at low temperatures. *Acta Cryst.* **9**, 671-677 (1956).
9. T. Slotwinski, J. Trivisonno, Temperature dependence of the elastic constants of single crystal lithium. *J. Phys. Chem. Solids* **30**, 1276-1278 (1969).
10. C. Fiolhais, J. P. Perdew, S. Q. Armster, J. M. MacLaren, M. Brajczewska, Dominant density parameters and local pseudopotentials for simple metals. *Phys. Rev. B* **51**, 14001-14011 (1995).
11. W. R. Tyson, W. A. Miller, Surface free energies of solid metals: Estimation from liquid surface tension measurements. *Surf. Sci.* **62**, 267-276 (1977).
12. F. R. d. Boer, R. Boom, W. C. M. Mattens, A. R. Miedema, A. K. Niessen, *Cohesion in metals: transition metal alloys*. (North-Holland, Amsterdam, 1988).
13. D. Gaissmaier, D. Fantauzzi, T. Jacob, First principles studies of self-diffusion processes on metallic lithium surfaces. *J. Chem. Phys.* **150**, 041723 (2019).
14. R. A. Silverman, W. Kohn, On the Cohesive Energy of Metallic Lithium. *Phys. Rev.* **80**, 912 (1950).
15. V. Schott, M. Fähnle, P. A. Madden, Theory of self-diffusion in alkali metals: I. Results for monovacancies in Li, Na, and K. *J. Phys.: Condens. Matter* **12**, 1171-1194 (2000).
16. W. Frank, U. Breier, C. Elsässer, M. Fähnle, First-Principles Calculations of Absolute Concentrations and Self-Diffusion Constants of Vacancies in Lithium. *Phys. Rev. Lett.* **77**, 518-521 (1996).
17. T. Krauskopf, B. Mogwitz, C. Rosenbach, W. G. Zeier, J. Janek, Diffusion Limitation of Lithium Metal and Li–Mg Alloy Anodes on LLZO Type Solid Electrolytes as a Function of Temperature and Pressure. *Adv. Energy Mater.* **9**, 1902568 (2019).
18. T. Oda, Y. Oya, S. Tanaka, W. J. Weber, Validation of potential models for Li2O in classical molecular dynamics simulation. *J. Nucl. Mater.* **367-370**, 263-268 (2007).
